# Determinants of attitude and intention towards private health insurance: a comparison of insured and uninsured young adults in Australia

**DOI:** 10.1186/s12913-021-06249-y

**Published:** 2021-03-19

**Authors:** Lisa Tam, Ellen Tyquin, Amisha Mehta, Ingrid Larkin

**Affiliations:** 1 School of Advertising, Marketing and Public Relations, Queensland University of Technology, 2 George Street, Brisbane, QLD 4000 Australia; 2 QUT Business School, Queensland University of Technology, 2 George Street, Brisbane, QLD 4000 Australia

**Keywords:** Australia, Private health insurance, Trust, Value, Young adults

## Abstract

**Background:**

Since the introduction in 1984 of Australia’s publicly-funded universal healthcare system, Medicare, healthcare financing has relied on a mix of public and private sources to meet the needs of the population (Sowa et al., Appl Health Econ Health Policy 15:31–41, 2018). However, in recent years, there has been a decline in the number of Australians choosing to purchase private health insurance (PHI), particularly within the young adult age group with the proportion of insurance customers aged 20 to 29 falling from 10.3 to 9.4% between 2012 and 2017 (Sivey, The Conversation, 2017). Young adults are critical to private health insurance funding models as their involvement offsets the drawdown by older adults (Dalzell and Borys, ABC News, 2019). While this issue is widely reported in the Australian media, few empirical studies have explored the factors that enable or constrain young adults’ enrolment in PHI.

**Methods:**

To address the scarcity of research about the motivational factors behind young adult decision-making, this study conducted a survey of 594 Australian young adults aged between 18 and 30 years. Within this age group, the survey sought an equal split of participants who were members and non-members of PHI schemes.

**Conclusion:**

The findings identified perceived value and trust in insurers as additional motivational factors alongside traditional measures of recognition of the problem and involvement in the problem. Differences between the insured and uninsured groups were identified which help to shape a more holistic understanding of the key motivational factors and barriers in relation to Australian young adults’ enrolment in PHI.

**Supplementary Information:**

The online version contains supplementary material available at 10.1186/s12913-021-06249-y.

## Background

Private health insurance (PHI) has been a significant social and policy issue that has received extensive coverage within the Australian media [1] primarily due to its cost [2] and complexity to comprehend [3]. Despite Australia’s publicly-funded universal healthcare scheme, Medicare, which all Australians have access to and most taxpayers contribute 2% of their taxable income to fund, PHI still remains an important component of healthcare funding [4]. According to official statistics released in March 2020, 43.80% of the Australian population was voluntarily enrolled in PHI [5]. However, there has been a downward trend. For example, between December 2019 and March 2020 there has been a 0.2 percentage point decrease in enrolment, with the largest net decrease (11,176 people) in the age group between 25 and 29 [5]. This presents a growing concern for both the Australian government and healthcare sector as young adults are critical to the PHI system because they improve the risk pool [6]. The departure of healthy young adults from the PHI system has the power to impact the wider Australian health system as it increases the premiums paid by older people for PHI and puts pressure back onto the public health system [7].

Existing research on PHI in Australia has been driven from an *economic* perspective, focusing on the effectiveness of government initiatives to incentivise and reduce barriers to enrolment in PHI [4, 8–11]. It is compulsory for most Australian taxpayers to pay an annual Medicare Levy to fund the public health system and single people earning above A$90,000 or families earning above A$180,000 have to pay an additional Medicare Levy Surcharge (MLS) if they do not have PHI. In addition to this MLS, individuals aged 31 or above have to pay a 2% Lifetime Health Cover loading on their health insurance premiums for every year they go without hospital cover [10]. Further incentive to enrol in PHI by the Australian government is offered through age-based rebates on PHI premiums (ranging from approximately 8 to 33%) to encourage early enrolment in PHI [9]. Despite these economic levers, the acceleration of dropouts in PHI (from 47.4% in 2015 to 43.8% in 2020) suggests that economics alone does not explain the full story of why individuals choose to enrol in PHI [5]. As such, there is a need to examine wider motivational factors and barriers to enrolling in and maintaining PHI [11].

With this backdrop, this study proposes to examine the factors that affect young adults’ attitude and behavioural intentions toward PHI. The purpose of this study is three-fold: (1) to explore the attitudinal and motivational differences between young adults (aged 18–30 years) with and without PHI; (2) to identify the factors that motivate those without PHI to enrol in PHI; and (3) to examine the factors that motivate those with PHI to cancel PHI. Specifically, this study will compare the two groups (i.e., those with and those without PHI) in terms of their health consciousness, perceptual variables (e.g., problem recognition, constraint recognition, involvement recognition, past experiences), trust, perceived value, attitude and intention to enrol/cancel. The examination of the dynamics of these factors will provide a more holistic framework that seeks to explain variations in attitude and behavioural intentions toward PHI.

## Factors affecting PHI choices in Australia

Research on PHI in Australia to date has focussed on three perspectives: (i) policy levers, and (ii) individual risk, and (iii) promotion of PHI. First, on the policy side, there has been a series of PHI policy reforms that sought to increase uptake in PHI as referenced above. A 2007 study found the Medicare Levy Surcharge, premium rebates and Lifetime Health Cover Loading all had a positive effect on the uptake of PHI, but the magnitude of the effects was not easily untangled [10]. Conversely, another study found that these reforms only benefited those who would have purchased PHI regardless of policy levers rather than encouraging those who did not intend to enrol [9].

Second, an individuals’ decision to purchase PHI has been examined from a risk perspective. Given that health insurers are prohibited from adjusting premiums based on consumer risk (e.g., pre-existing conditions), a positive correlation between insurance uptake and expected claims can be expected, however data has shown that those with PHI actually spent fewer nights in hospitals [12]. Even though it was expected that individuals with poor health were more likely to purchase PHI due to their anticipation of the need of medical care, they were outnumbered by healthier consumers who had higher risk aversion [12].

Third, studies have begun to explore how organisations promoted PHI. A study of insurers’ websites found that health insurers promoted choice and healthy lifestyles as the benefits of PHI, suggesting this positioning was an effective marketing strategy [13]. Furthermore, a study of Australian health consumers found that consumers’ choices are not grounded in their individual experiences of the system but their futuristic expectations of benefit and whom they trust to help them minimise risk [14]. In the United States, factors including perceived health status, perceived value, perceived need, socioeconomic status and ethnicity significantly affected whether U.S. young adults would enrol in PHI and that there was a combination of factors that affected their choices [15].

Despite changes in policies that seek to encourage Australians into the PHI system and remove pressure on the public system and an expectation that due to risk aversion consumers would be motivated to maximise PHI utility [13], many Australians still choose to ‘ditch’ or refuse to enrol in PHI. This environment surrounding the PHI system points to the need to explore a new question. Instead of focusing on the economic reasons for why people, especially young people, do *not* take out PHI, we need to examine the motivational factors and barriers that explain young adults’ attitude and behavioural intentions toward PHI amongst those who have and who do not have PHI. Those with and without PHI may not use the same decision-making models. As such, this study seeks to contribute to knowledge on this topic by reviewing motivational factors that may explain young adults’ attitude and behavioural intentions toward PHI. This study will focus on young adults aged 18–30 in Australia because of their importance in balancing the risk pool [6]. Particularly, the following variables are examined and explained in the following sub-sections: health consciousness, perceptual variables (i.e., problem recognition, involvement recognition, constraint recognition, past experiences), trust, perceived value and attitude and intention to purchase/cancel PHI.

### Health consciousness

Individuals who have high health consciousness have been found to take greater responsibility for protecting their own health by complying with health-related recommendations [16], and adopting healthy behaviours [17, 18] such as maintaining a healthy diet [19, 20] and getting regular exercise [21]. Existing literature in health communication has found that individuals with higher levels of health consciousness (i.e., the extent to which health concerns are integrated into a person’s daily activities) are more likely to display intentions to search for health-related information [22, 23]. These studies have also shown that these individuals actively seek out health information and use more information sources than those who are not health-oriented [17]. Given the focus of current and future-oriented health behaviours noted in studies around health consciousness, this study proposed the following hypothesis:

- **H1o:** Australian young adults with and without PHI show no statistical difference in health consciousness.

### Perceptual factors

Existing research on PHI has examined individuals’ choices as being confined by policies, their individual evaluations of risk [14] and the maximisation of utility [24]. However these perspectives are based on decision-making theories that assume perfect and equal knowledge amongst individuals who maximise utility/satisfaction.

However, communication theories [24–26] criticise the assumption of perfect knowledge, arguing that knowledge and information is neither free nor given in decision situations. Instead these communication-based theories proport that when confronting a problem, individuals become engaged in communicative behaviours (e.g., information acquisition and transmission) that help them obtain the information and knowledge in order to make a decision. In the context of health, four variables in the Situational Theory of Problem Solving - problem recognition, constraint recognition, involvement recognition and referent criterion - have been used to predict individuals’ engagement in health-related communicative behaviours [27–31]. The theory presents the argument that when individuals perceive the presence of a problem (e.g., the need for PHI), feel connected to the problem, and see few obstacles in solving the problem, they will be engaged in behaviours to solve the problem. It has been previously used to guide understanding on motivations to act on health issues such as weight loss and organ donation [26, 32, 33].

Building on the Situational Theory of Problem Solving [26], this study proposes to examine four variables as possible perceptual factors that differentiate young adults with and without PHI. First, problem recognition is defined as an individual’s perceptions of a discrepancy between the expected state and the experiential state (e.g., perceptions of lack of PHI as a problem). Second, involvement recognition is defined as the connection between oneself and the problem (e.g., one’s being personally affected by not having PHI). Third, constraint recognition refers to the perceptions of obstacles that limit one’s ability to solve the problem (e.g., the lack of resources to solve the problem). Lastly, referent criterion, generally referred to as past experiences that guide one’s approaches to solving the problem, is operationalised as one’s past experiences with PHI. Accordingly, the following hypothesis is proposed:

- **H2o:** Australian young adults with and without PHI show no statistical difference in their (a) problem recognition, (b) constraint recognition, (c) involvement recognition and (d) past experiences with PHI.

### Perceived value

Previous literature indicates that people are more likely to invest in health insurance if they perceive the benefits exceed the out-of-pocket costs [15]. A U.S. study found that the rising cost of insurance premiums is a major reason why so many young adults do not purchase PHI [15]. This sentiment can be reflected within Australia, with significant rises in premium costs [34]. While cost is not an element that communicators are able to change, the concept of perceived value is worth addressing. Currently it can be argued that many young adults’ perceived value of PHI does not outweigh its cost. In support of this, the U.S. study found that while individuals’ perception of health insurance’s value (worth or not worth the cost) was not significantly correlated with the likelihood of having health insurance in their 2005 sample, perceived value was a statistically significant variable in 2008 [15]. Thus, the following hypothesis is proposed:

- **H3o:** Australian young adults with and without PHI show no statistical difference in their perceived value of PHI.

### Trust

Trust has been the subject of many empirical studies and has been found to be an important factor in cultivating long-term, positive relationships between an organisation and its strategic stakeholders [35], enhancing customer loyalty [36], gaining positive word-of-mouth recommendations [37] and increasing purchase intention [38]. In the PHI context, an exploratory, qualitative study investigated the motivations for the uptake of PHI in young Australians [14]. It noted that reasons for health insurance decision-making did not reflect a “rational or calculative” approach (p. 399). The authors found that young adults rely less on evidence (such as their PHI contract or previous experience) than they do on trust in the system [14]. As a result the paper suggests people do not calculate the possibilities of ill health, or weigh up the costs and benefits of private and public provision of health care [14], instead they rely on more of an emotive response to purchasing PHI. Building on the findings of this study, we intend to incorporate trust in PHI providers as a key element of this study to determine its potential impact on PHI decision making with the following hypothesis:

- **H4o:** Australian young adults with and without PHI show no statistical difference in their trust in insurance companies.

### Attitude

While the perceptual factors (i.e., problem recognition, constraint recognition, involvement recognition, past experiences), perceived value and trust have been identified as possible factors that characterise the differences between those with and without PHI in previous research, this study follows other behaviour-related literature in positing the dynamics amongst perceptions, attitude and behavioural intentions/behaviours [39]. The Theory of Planned Behaviour suggests that there are relations among beliefs, attitude, intentions, and behaviours [40]. Attitude is defined as “the degree to which a person has a favourable or unfavourable evaluation or appraisal of the behaviour in question” (p. 188), and the formation of attitude is dependent upon perceptual variables and beliefs which are associated with the intentions of performing the behaviours [41]. Thus, this study proposes that those with and without PHI would differ in terms of their attitude and that the identified variables would affect individuals’ attitude toward PHI.

- **H5o:** Australian young adults with PHI and without PHI show no statistical difference in their attitude toward PHI.
- **H6o:** (a) Problem recognition, (b) constraint recognition, (c) involvement recognition, (d) perceived value, and (e) trust have no statistically significant relationships with attitude toward PHI.

### Intention to enrol/cancel PHI

Following the models which explain the dynamics amongst perceptions, attitude and behavioural intentions, this study posits that there are relationships between past experiences and behavioural intentions to cancel (for those with PHI) and to enrol (for those without PHI). This proposition is based on theory that suggests positive attitude toward a behaviour strengthens an individual’s intention to perform the behaviour under consideration [40]. Accordingly this study proposes that attitude will have a positive effect on intention to enrol (for those without PHI) and a negative effect on intention to cancel (for those with PHI). The following hypotheses are proposed.

- **H7o:** Past experiences have no associations with (a) intention to enrol (for those without PHI) and (b) intention to cancel (for those with PHI).
- **H8o:** Attitude has no associations with (a) intention to enrol (for those without PHI) and (b) intention to cancel (for those with PHI).

**Fig. 1:**
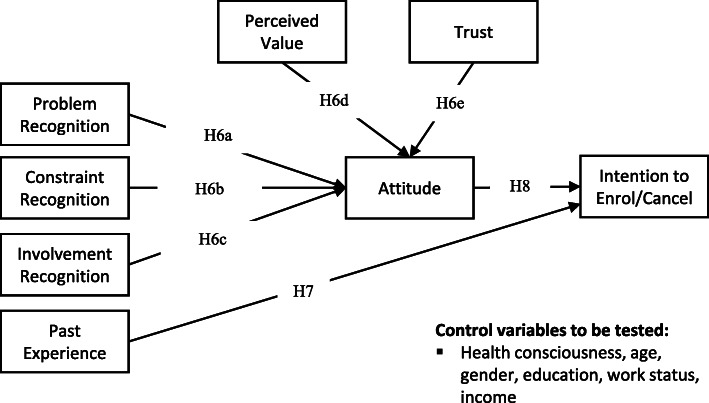
Hypothesised model to be tested

Figure 1 shows a proposed model with Hypotheses 6–8.

## Methods

### Measures

An online questionnaire was developed to explore the dynamics of the variables and to test the hypotheses. First, the survey items were all adopted from existing studies, including health consciousness (five items) [22], the four perceptual variables (i.e., four items for problem recognition, constraint recognition, involvement recognition and past experience each) [26], perceived value (“I do not think I can get my value for money from private health insurance”) [15], trust (six items) [42], attitude (four items) and behavioural intentions (“I intend to cancel my private health insurance” or “I intend to sign up for private health insurance in the future”) [40].

### Data collection and demographics

Upon approval from the University’s ethics committee, Survey Sampling International (SSI, now known as Dynata) was commissioned to recruit two groups of Australian young adults (aged 18–30 years) to complete a 15-min online questionnaire. Participants were recruited to be representative of the Australian population in terms of age and gender in the selected age range. The 18–30 age range was selected because their premiums were not affected by the Lifetime Health Cover Loading [10]. Despite their importance in improving the risk pool of PHI, there are no policy incentives (e.g., increased costs) which affect their intention to enrol or cancel. The downward trend in this age group’s enrolment in PHI warrants further investigation into other factors which affect their motivations to enrol or to cancel PHI [5]. The participants were incentivised for their time based on their agreements with SSI. A total of 583 valid questionnaires were received in April 2018. Of this total sample, 282 (48.4%) respondents had PHI and 301 (51.6%) respondents did not have PHI. Table 1 shows a breakdown of demographics of the two groups.

**Table 1:** Demographics of the respondents

	With PHI ***n*** = 282 (48.4%)	Without PHI ***n*** = 301 (51.6%)	Statistical difference
**Gender**			
Male	130 (46.1%)	116 (38.5%)	No difference due to sample requirements
Female	151 (53.5%)	184 (61.1%)	
Prefer not to say	1 (.4%)	1 (.3%)	
**Age**			
18–20	30 (10.6%)	46 (15.3%)	No difference due to sample requirements
21–25	222 (78.7%)	106 (35.2%)	
26–30	30 (10.6%)	149 (49.5%)	
**Education**			
Primary school	2 (.7%)	3 (1%)	F = 6.8, df = 5, *p* < .001
High school	79 (28%)	131 (43.5%)	
Undergraduate	79 (28%)	68 (22.6%)	
Degree, trade, or certificate	78 (27.7%)	71 (23.6%)	
Master or doctorate	42 (14.9%)	16 (5.3%)	
Other	2 (.7%)	12 (4%)	
**Work Status**			
Full time	140 (49.6%)	89 (29.6%)	F = 12.816, df = 5, *p* < .001
Part time	50 (17.7%)	35 (11.6%)	
Casual	17 (6%)	30 (10%)	
Student	44 (15.6%)	41 (13.6%)	
Unemployed	25 (8.9%)	88 (29.2%)	
Not applicable	6 (2.1%)	18 (6%)	
**Income**			
< AUD$18,200	49 (17.4%)	108 (35.9%)	F = 13.142, df = 5, *p* < .001
$18,200–$37,000	36 (12.8%)	58 (19.3%)	
$37,001–$87,000	107 (37.9%)	86 (28.6%)	
$87,001–$180,000	45 (16%)	12 (4%)	
> $180,00	16 (5.7%)	2 (.7%)	
Undisclosed	29 (10.3%)	35 (11.6%)	

Because SSI was instructed to obtain comparable samples by age and gender for those with and without PHI, the two groups did not differ in terms of age and gender. But for educational attainment, there is a significant difference (F = 6.8, df = 5, *p* < .001) with a post-hoc test showing significant difference between those with high school education and masters and doctorate (*p* < .001). The latter is more likely to have PHI. Significant differences were also found for paid work status (F = 12.816, df = 5, *p* < .001). The post-hoc tests showed that the “unemployed” group was the least likely to have PHI and was significantly different from those with full-time work (*p* < .001) and part-time work (*p* < .001) who were the most likely to have PHI. Lastly, significant differences were also found for income (F = 13.142, df = 5, *p* < .001). The post-hoc tests showed a positive relationship between levels of income and PHI status. The higher the income, the higher the likelihood of having PHI.

### Data analysis

Before proceeding with hypotheses testing, items for constraint recognition which were positively worded were first reverse-coded. Then, Exploratory Factor Analysis (EFA) using Maximum Likelihood with Promax Rotation [43] was conducted on the survey items used for each variable (which is evaluated using a five-point Likert scale from “strongly disagree” to “strongly agree”) using SPSS (Statistical Package for the Social Sciences) Version 25. This ensures the latent structure of the observed variables [44], which was needed because the items were adopted from existing research studies but were applied to a PHI context. Table 2 shows the factor loadings, the mean standard deviation (SD) and standard error (SE) of the mean for each item that was retained. Items with factor loadings of less than .6 were removed. The factor loadings of retained items ranged from .634 to .904. The Cronbach alpha (α) ranged from .743 to .931. The Kaiser-Meyer-Olkin (KMO) measure of adequacy for each variable ranged from .693 to.904. The Bartlett’s test of sphericity was significant for all variables. The variance explained ranged from 61.53 to 78.85%. The eigenvalues of all retained factors were all above 1. The EFA indicated that the retained items were adequate to explain the latent variables [45]. After confirming the dimensionality of each construct, a Confirmatory Factor Analysis (CFA) was conducted for each construct to identify the factor weights for creating the composite scores for testing the hypotheses on SPSS and AMOS. Subsequently, Hypotheses 1–5 were tested using t-tests and ANOVAs to test the differences between those with and without PHI. Structural Equation Modelling (SEM) was performed to test Hypotheses 6–8.

**Table 2:** Factor loadings, mean, standard deviation, and standard error of the mean for each survey item (α = Cronbach’s alpha, KMO = Kaiser-Meyer-Olkin, M = mean, SD = standard deviation, SE = standard error)

Variable	Survey Item	Factor Loadings	Mean	SD	SE
**Health consciousness** **α** = .757 **KMO** = .773 **Variance explained** = 61.53%	I do everything I can to stay healthy.	Removed			
	Living life in best possible health is important to me.	.803	3.96	.892	.037
	I actively try to prevent diseases and illnesses.	.699	3.89	.912	.038
	Eating right, exercising, and taking preventive measures will keep me healthy for life.	.634	4.03	.875	.036
	My health depends on how well I take care of myself.	Removed			
**Problem recognition** **α** = .881 **KMO** = .804 **Variance explained** = 73.76%	I think not having private health insurance is a problem.	.839	3.13	1.172	.049
	I am concerned about those who do not have private health insurance.	.848	2.86	1.182	.049
	Something needs to be done to encourage Australians to purchase private health insurance.	.783	3.23	1.152	.048
	I recognise the importance of having private health insurance.	.752	3.45	1.167	.048
**Constraint recognition** **α** = .743 **KMO** = .782 **Variance explained** = 63.31%	I feel capable of protecting myself by having private health insurance.	Removed			
	There are no barriers stopping me from having private health insurance.	.682	2.93	1.29	.054
	I feel confident about choosing the right private health insurance plan for myself.	.846	3.17	1.17	.048
	It is easy to purchase private health insurance.	.705	3.25	1.15	.048
**Involvement recognition** **α** = .910 **KMO** = .803 **Variance explained** = 78.75%	Not having private health insurance can affect me personally.	.803	3.29	1.145	.047
	Not having private health insurance can have consequences for me and those I care about.	.799	3.32	1.147	.048
	Not having private health insurance can threaten my health.	.904	2.98	1.193	.049
	My health can be affected if I do not have private health insurance.	.871	2.94	1.211	.050
**Past Experiences** **α** =. 871 **KMO** = .768 **Variance explained** = 72.09%	My health can be affected if I do not have private health insurance.	.853	2.46	1.314	.054
	Not having private health insurance has caused me problems in the past.	.816	2.58	1.288	.053
	I have dealt with problems caused by not having private health insurance in the past.	.782	2.83	1.299	.054
	My past experiences have taught me the importance of private health insurance.	.712	2.73	1.290	.053
**Trust** **α** = .931 **KMO** = .904 **Variance explained** = 74.4%	Insurance companies treat their customers fairly and justly.	.931	3.04	1.05	.043
	Whenever insurance companies make important decisions, they are concerned about their customers.	.884	2.86	1.12	.046
	Insurance companies can be relied on to keep their promises for customers.	.886	2.93	1.109	.046
	Insurance companies take the opinions of their customers into account when making decisions.	.857	2.97	1.122	.046
	Insurance companies have the ability to accomplish when they say they will do for their customers.	.687	3.21	1.045	.043
	Insurance companies will do what they say they will do for their customers.	.841	2.99	1.045	.043
**Attitude** **α** = .817 **KMO** = .693 **Variance explained** = 73.43%	It is good to have private health insurance.	.709	3.74	1.015	.042
	It is dangerous not to have private health insurance.	.724	2.98	1.181	.049
	It is worth spending money on private health insurance.	.898	3.26	1.139	.047
	It is worth ensuring that private health insurance has adequate coverage.	Removed			

### Results

Table 3 shows the results from the hypothesis testing.

**Table 3:** Results from the hypotheses tested

Hypothesis	Variable	Estimate	df	***p***-value	Result
***Comparisons between those with and without PHI***					
H1o	Health consciousness	t = 2.857	581	.004	rejected
H2o	(a) Problem recognition	t = 3.37	581	.000	rejected
	(b) Constraint recognition	t = 11.86	581	.000	rejected
	(c) Involvement recognition	t = 11.20	581	.000	rejected
	(d) Past experience	t = 9.56	579	.000	rejected
H3o	Perceived value	t = −.234	576	.020	rejected
H4o	Trust	t = 4.72	574	.000	rejected
H5o	Attitude	t = 12.87	581	.000	rejected
***Hypotheses tested for those without PHI***					
H6o	(a) Problem recognition – Attitude	β = .388	–	.000	rejected
	(b) Constraint recognition – Attitude	β = .006	–	.881	fail to be rejected
	(c) Involvement recognition – Attitude	β = .320	–	.000	rejected
	(d) Percieved value – Attitude	β = −.184	–	.000	rejected
	(e) Trust – Attitude	β = .140	–	.000	rejected
H7o	Past experience – intention to enrol	β = .186	–	.000	rejected
H8o	Attitude – intention to enrol	β = .482	–	.000	rejected
***Hypotheses tested for those with PHI***					
H6o	(a) Problem recognition – Attitude	β = .396	–	.000	rejected
	(b) Constraint recognition – Attitude	β = .054	–	.283	fail to be rejected
	(c) Involvement recognition – Attitude	β = .197	–	.000	rejected
	(d) Percieved value – Attitude	β = −.106	–	.015	rejected
	(e) Trust – Attitude	β = .221	–	.000	rejected
H7o	Past experience – intention to cancel	β = .082	–	.155	fail to be rejected
H8o	Attitude – intention to cancel	Β = -.327	–	.000	rejected

H1 examined whether those with and without PHI are significantly different in terms of their health consciousness (measured using three formative items with a five-point Likert scale). T-test results showed statistical differences between the two groups for health consciousness (t = 2.857, df = 581, *p* < .01); the PHI group has higher health consciousness (M = 4.05, SD = .68, SE = .041) and those without PHI had lower health consciousness (M = 3.88, SD = .78, SE = .045). The null hypothesis for H1 is rejected.

H2 examined the two groups’ differences in terms of their perceptual variables: problem recognition (t = 3.37, df = 581, *p* < .001), constraint recognition (t = 11.86, df = 581, *p* < .001), involvement recognition (t = 11.2, df = 581, *p* < .001) and past experiences (t = 9.56, df = 579, *p* < .001). The null hypothesis for H2 is rejected. The two groups also differed in perceived value (t = .234, df = 576, *p* < .05) and trust (t = 4.72, df = 574, *p* < .001), so the null hypotheses for H3 and H4 were rejected. The null hypothesis for H5 was also rejected as the two groups differed in their attitude toward PHI (t = 12.87, df = 581, *p* < .001).

**Fig. 2:**
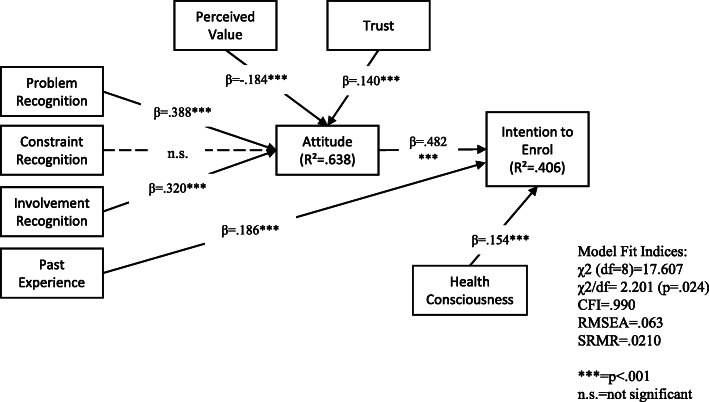
Structural model for the group without PHI

Results from Structural Equation Modelling are shown in Fig. 2 for the group without PHI. For the group without PHI, problem recognition (β = .388, *p* < .001), involvement recognition (β = .320, *p* < .001), perceived value (β = −.184, *p* < .001) and trust (β = .140, *p* < .001) were all associated with attitude, but the null hypothesis for H6b failed to be rejected as constraint recognition had no relationships with attitude. Past experiences (β = .186, *p* < .001) and attitude (β = .482, *p* = .024) positively predicted intention to enrol. Health consciousness was found to have a significant relationship with intention to enrol (β = .154, *p* < .001) and was thus, added as a control variable to the study. The model fit was acceptable (χ2 = 17.607, df = 8, χ2/df = 2.201, *p* = <.05, CFI = .990, RMSEA = .063, SRMR = .0210) based on Hu and Bentler’s (1999) cut-off criteria for fit indices (χ2/df < 3, CFI > .95, RMSEA<.06). The model predicts 40.6% of individuals’ intention to enrol.

**Fig. 3:**
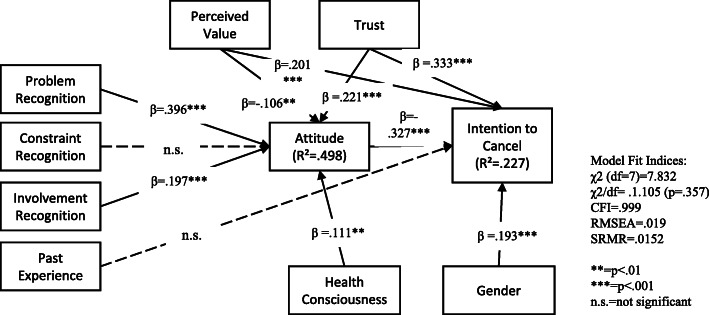
Structural model for the group with PHI

Results from Structural Equation Modelling are shown in Fig. 3 for the group with PHI. Problem recognition (β = .396, *p* < .001), involvement recognition (β = .197, *p* < .001), perceived value (β = −.106, *p* < .05) and trust (β = .221, *p* < .001) were all associated with attitude, but the null hypothesis for H6b also failed to be rejected for this group as constraint recognition had no relationships with attitude. Past experience had no relationship with intention to cancel but attitude (β = −.327, *p* < .001) had a negative relationship with intention to cancel. Health consciousness was positively associated with attitude (β = .111, *p* < .01) and gender was associated with intention to cancel (β = .193, *p* < .001). Females were less likely to cancel PHI. These variables were added to the model as control variables. The model fit was acceptable (χ2 = 7.832, df = 7, χ2/df = 1.105, *p* = .357, CFI = .999, RMSEA = .019, SRMR = .0152) based on Hu and Bentler’s (1999) cut-off criteria for fit indices (χ2/df < 3, CFI > .95, RMSEA<.06). The model predicts 22.7% of individuals’ intention to cancel PHI.

## Discussion

The findings from this study provide a more holistic picture of the differing motivational factors and barriers between young adults with PHI and those without PHI beyond the economic perspective. Reflecting on the differences between the two groups, it is of note that those with PHI are generally higher in health consciousness which corresponds to existing findings [24] that healthier individuals are more risk averse. This study also identified perceived value and trust in insurance companies as significant factors affecting attitude towards PHI for both groups. Combined these findings point to the need to not only use demographic variables (e.g., socioeconomic factors like education and income) to understand young adults’ intentions to enrol or cancel PHI. As highlighted in the models above, health consciousness, perceptual variables, perceived value and trust also should be considered when exploring this decision-making process.

This study has examined the motivational factors that affected young adults’ attitude and behavioural intentions towards PHI. The findings have several implications. In comparing young adults with and without PHI, this study found that there are demographic differences (e.g., education, work status and income) that affected attitude and intention that policymakers cannot easily influence. But the differences in other factors including health consciousness, problem recognition, involvement recognition and constraint recognition value can be managed through communication intervention campaigns to promote health beliefs, healthy activities, and benefits of PHI. As for trust and perceived value, it requires responses from PHI providers. On one hand, policies can be adjusted and communication interventions can be implemented with intent to increase the attractiveness of PHI. On the other hand, the lack of trust in PHI providers can negatively affect attitude.

The structural model for the group without PHI shows that past experiences and health consciousness directly increase intention to enrol, whereas problem recognition, involvement recognition, trust and perceived value affect attitude. Policymakers and PHI providers should consider how the promotion of health consciousness amongst the population could also improve PHI uptake. On the other hand, the structural model for the group with PHI shows that past experiences do not affect intention to cancel but gender does. Males show higher intention to cancel than females. With females identified as being more risk-averse, their influential roles in affecting other household members (e.g., males) decisions on PHI could be significant. Lastly, from a policy perspective, although existing studies found that policy changes (e.g., rebates and tax implications) are significant in predicting PHI uptake, it is noteworthy that these policies may not entirely benefit young adults. For example, the Lifetime Health Cover Loading in Australia does not apply to those under the age of 31 [10]. Thus, factors that contribute to an individual’s intention to enrol and intention to cancel should be explored more comprehensively. Perceptions of the other significant factors (including problem recognition, involvement recognition, trust and perceived value) can be improved with investments in communication interventions and organisational actions.

### Limitations

As in any research, there are limitations in the study. First, this dataset is cross-sectional and was collected in April 2018. Future studies should consider collecting data at two different times to explore how environmental factors such as policy changes or changes in the economic environment may affect the hypotheses tested. Second, two variables, namely perceived value and behavioural intention, were measured using one item. To ensure reliability and validity, future studies should use multiple items. Third, while this study has examined a number of factors that affected decisions, there could be other factors, such as parental influence, which have not been explored. Although the model for the group without PHI can explain 40.6% of respondents’ intention to enrol, the model for the group with PHI only explains 22.7% of respondents’ intention to cancel. As such, there could be other determinants explaining the intention to cancel. Lastly, because the sample was representative of the Australian population by the selected age range, this study was not able to identify whether age was related to PHI status. Future studies could investigate whether the cost of PHI could be a barrier for younger adults because of their relative income. These studies could consider price floors and ceilings that are perceived as affordable by young adults and older adults, noting that that PHI premium increases are regulated by PHI insurance laws in Australia.

## Conclusion

Although the Australian government has implemented economic incentives to encourage PHI uptake, many of these incentives do not benefit young adults. Young adults aged 18 to 30 are critical to improving the risk pool of PHI, but the industry has continued to face the challenge of failing membership among young adults. This has raised concerns about the worsening affordability of PHI and the long-term sustainability of the industry. While existing studies have mostly focussed the effectiveness of economic incentives in improving uptake, this study proposed that for young adults as a demographic group, it is important to understand the motivational factors and barriers to PHI beyond the economic perspective. By comparing the differences between insured and uninsured young adults, this study found that factors including education, work status and income could affect PHI uptake. As these factors cannot be changed by policymakers, the findings proposed that promoting health consciousness amongst young adults and improving trust in insurance companies and perceived value could potentially improve PHI uptake and reduce PHI dropout amongst young adults.

## Supplementary Information

**Additional file 1.**

## Data Availability

All data generated or analysed during this study are included in this published article.

## Abbreviation

PHI: Private Health Insurance
