# Supplementary material for: Determinants of attitude and intention towards private health insurance: a comparison of insured and uninsured young adults in Australia
Source: BMC Health Serv Res. 2021 Mar 19;21:246. doi: 10.1186/s12913-021-06249-y (PMC7977238; doi:10.1186/s12913-021-06249-y)
Supplement: Supplementary file 1 — Additional file 1. [file 12913_2021_6249_MOESM1_ESM.docx]

**Questionnaire**

**Scale used:** strongly disagree (1) to strongly agree (5)

**Health coconsciousness**

- I do everything I can to stay healthy.*
- Living life in best possible health is important to me.
- I actively try to prevent diseases and illnesses.
- Eating right, exercising, and taking preventive measures will keep me healthy for life.
- My health depends on how well I take care of myself.*

**Problem recognition**

- I think not having private health insurance is a problem.
- I am concerned about those who do not have private health insurance.
- Something needs to be done to encourage Australians to purchase private health insurance.
- I recognise the importance of having private health insurance.

**Constraint recognition**

- I feel capable of protecting myself by having private health insurance.*
- There are no barriers stopping me from having private health insurance.
- I feel confident about choosing the right private health insurance plan for myself.
- It is easy to purchase private health insurance.

**Involvement recognition**

- Not having private health insurance can affect me personally.
- Not having private health insurance can have consequences for me and those I care about.
- Not having private health insurance can threaten my health.
- My health can be affected if I do not have private health insurance.

**Past experiences**

- My health can be affected if I do not have private health insurance.
- Not having private health insurance has caused me problems in the past.
- I have dealt with problems caused by not having private health insurance in the past.
- My past experiences have taught me the importance of private health insurance.’

**Perceived value**

- I do not think I can get my value for money from private health insurance.

**Trust in insurance companies**

- Insurance companies treat their customers fairly and justly.
- Whenever insurance companies make important decisions, they are concerned about their customers.
- Insurance companies can be relied on to keep their promises for customers.
- Insurance companies take the opinions of their customers into account when making decisions.
- Insurance companies have the ability to accomplish when they say they will do for their customers.
- Insurance companies will do what they say they will do for their customers.

**Attitude**

- It is good to have private health insurance.
- It is dangerous not to have private health insurance.
- It is worth spending money on private health insurance.
- It is worth ensuring that private health insurance has adequate coverage.*

**Behavioural intention (for young adults with PHI)**

- I intend to cancel my private health insurance.

**Behavioural intention (for young adults without PHI)**

- I intend to sign up for private health insurance in the future.

*Items removed from the analysis due to low factor loadings.
